# Unveiling the hidden connection: the blood-brain barrier’s role in epilepsy

**DOI:** 10.3389/fneur.2024.1413023

**Published:** 2024-08-14

**Authors:** Jinkun Han, Ying Wang, Penghu Wei, Di Lu, Yongzhi Shan

**Affiliations:** Department of Neurosurgery, Xuanwu Hospital Capital Medical University, Beijing, China

**Keywords:** epilepsy, blood-brain barrier, angiogenesis, inflammation, biomarkers

## Abstract

Epilepsy is characterized by abnormal synchronous electrical activity of neurons in the brain. The blood-brain barrier, which is mainly composed of endothelial cells, pericytes, astrocytes and other cell types and is formed by connections between a variety of cells, is the key physiological structure connecting the blood and brain tissue and is critical for maintaining the microenvironment in the brain. Physiologically, the blood-brain barrier controls the microenvironment in the brain mainly by regulating the passage of various substances. Disruption of the blood-brain barrier and increased leakage of specific substances, which ultimately leading to weakened cell junctions and abnormal regulation of ion concentrations, have been observed during the development and progression of epilepsy in both clinical studies and animal models. In addition, disruption of the blood-brain barrier increases drug resistance through interference with drug trafficking mechanisms. The changes in the blood-brain barrier in epilepsy mainly affect molecular pathways associated with angiogenesis, inflammation, and oxidative stress. Further research on biomarkers is a promising direction for the development of new therapeutic strategies.

## Graphical abstract

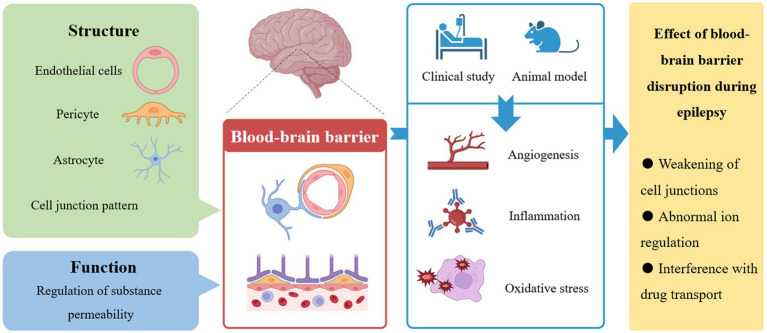

## 1 Introduction

Epilepsy is a neurological disorder characterized by abnormal neuronal discharges, and often clinically manifests as convulsions or disturbance of consciousness (1). Epilepsy affects at least 65 million people worldwide and is the fourth most prevalent neurological disorder (2, 3). Although the prevalence of epilepsy has aroused great enthusiasm for epilepsy research among scientists, the pathogenic mechanism in 50% of epilepsy cases remains unknown (4). This gap in knowledge is also an important reason for the currently high proportion of drug-resistant epilepsy [notably, 38% in temporal lobe epilepsy (5)].

Abnormalities in cerebral vessels, a closely related structure to neural tissue, are necessarily associated with neurological diseases (6–8), including epilepsy (9). Alterations in microvascular networks can be observed in the sclerotic hippocampus of temporal lobe epilepsy patients (10). In 60% of patients with epilepsy, symptoms are initiated by structural causes, such as traumatic brain injury (TBI) or stroke (1, 11), which are generally considered change blood-brain barrier (BBB) permeability. Indeed, discussions about the role of the blood-brain barrier in epilepsy have continued for decades (12, 13). Studies have shown that disruption of the BBB can cause seizures or aggravate epilepsy (14, 15), and conversely, progression of epilepsy can further disrupt the BBB (16–18). We discuss the structure of the BBB and its role in epilepsy in the following sections. In addition, we discuss the discovery of biological targets associated with the BBB to provide a reference for further improvement of epilepsy treatment strategies in the future.

## 2 Establishment of the blood-brain barrier

### 2.1 Structure of the BBB

In the nervous system, precise signaling activity places high demands on microenvironment stability. This is the significance of the existence of the BBB (6, 19). Ehrlich (20) discovered that hydrophilic dyes injected into the blood circulation stain peripheral organs but not the spinal cord and brain, thus confirming the existence of a special interface between the central nervous system and blood for the first time. Stern (21) postulated that there was a barrier between blood and neuronal tissue and coined the name blood-brain barrier. The BBB is necessary for normal activity in the central nervous system (CNS). All organisms with a well-developed CNS have a well-established BBB (22). In adults, capillary endothelial cells (ECs) are the main component of the BBB and constitute a blood-brain exchange interface area of 12 to 18 cm^2^. Even with such a large contact area, capillary ECs in the CNS, unlike those in most peripheral organs, lack small pores that would allow polar solutes to pass through the barrier because of the presence of tight junctions (TJs). TJs are composed of different transmembrane proteins, including junctional adhesion molecules (JAMs), claudins, and membrane-associated guanylate kinase (MAGUK)-like proteins, and have the effect on plugging gaps between ECs. Therefore, TJs restrict the free transportation of polar solutes such as ions and solutes through paracellular diffusion channels by imparting the high transendothelial electrical resistance (TEER) of the BBB (23). In addition to TJs, interendothelial gap also features adherens junctions (AJs) and related proteins functioning as junctional complexes, which are involved in the maintenance of BBB permeability, cell adhesion and angiogenesis (24). Loss of AJs can cause decreased BBB integrity and increased permeability.

In contrast to peripheral capillaries, special structures such as pericytes, basal lamina, and astrocyte end feet cover the periphery of the vessels around capillaries in the CNS. Pericytes are located outside ECs and embedded in the vascular basement membrane, which plays an important role in regulating angiogenesis, extracellular matrix deposition, and immune cell infiltration in the CNS. The basement membrane covers the adventitial surface of ECs and extends around capillaries but separates postcapillaries at venules. This structure creates a perivascular space that facilitates drainage of cerebrospinal fluid (CSF) for immunosurveillance (25). Astrocytes can polarize neuronal processes or perivascular cellular processes and participate in the regulation of blood flow (26). Pericytes and the end feet of astrocytes are connected (27, 28), which becomes the anatomical basis for their functional relationship during development and inflammation (29). Studies have confirmed the involvement of astrocytes and pericytes in the deposition of basal lamina during angiogenesis (23, 30) and the developmental localization and polarization of ATP-binding transporters on endothelial cells (31). In addition, CNS-associated macrophages are also located between astrocyte terminals and parenchymal vessels, where they extend into the perivascular space and participate in immune processes by phagocytosing debris (32).

Recent research has demonstrated the significant contribution of microglia in the maintenance of blood-brain barrier (BBB) function. Microglia are involved in the stabilization of intracranial neovascularization, facilitation of vasoconstriction, and mediation of blood flow signaling upon activation (33–37). Various factors have the potential to impede BBB function and compromise BBB integrity by influencing microglial activity (38, 39). Notably, microglia play a role in synaptic remodeling and contribute to brain development within the nervous system (40, 41).

### 2.2 Permeability and barrier function of the BBB

The BBB acts as a barrier between the brain and the blood that connects external substances, and its function is achieved by limiting or allowing different substances to cross. Compared with that of peripheral vessels, BBB permeability is much less, generally allowing passive diffusion of lipophilic molecules less than 500 Da and few hydrogen-bonded donors and/or acceptors under physiological conditions (42, 43). In addition to mass, lipophilicity, and the presence of hydrogen bonds, the charge and polar surface area of the compound can also influence the associated BBB permeability (6, 44). These factors contribute to the inability of 98% of drugs to cross the BBB (45) while small, lipophilic, uncharged antiepileptic drugs can easily cross the BBB (46). In addition, a few antiepileptic drugs, such as the short branched-chain fatty acid valproate, can also enter the brain through carrier-dependent active transport (47, 48). Different ion channels and transporters at the BBB can complete the transmembrane transmission of charges, thereby helping to maintain synaptic signaling activity and ion stability in the internal environment. At the BBB, levels of potassium (K^+^), magnesium (Mg^2+^), and calcium (Ca^2+^) ions are differently regulated. Potassium ions are present in mammalian plasma at concentrations of approximately 4.5 mMol. In CSF, however, the potassium concentration is approximately 2.5–2.9 mMol and is not affected by external factors such as food consumption, exercise, pathological conditions, etc. (49, 50). Selective and region-specific expression of some substance transporters in ECs ensures polarization of ECs (51). In addition, the BBB is involved in completing the regulation of neurotransmitter distribution in the CNS by physically separating central and peripheral nerves. Neurotransmitters such as glutamate are released in large amounts into the cerebral CSF during hypoxia in ischemic stroke and can lead to neurotoxic damage (52). Therefore, the permeability of the BBB also plays a significant protective role in the central nervous system. On the one hand, the BBB prevents macromolecules such as albumin, prothrombin, and plasminogen from entering the CNS, preventing apoptosis and damage to neural tissue (53, 54). On the other hand, the BBB acts as a barrier to prevent invasion of the CNS by toxins in the blood, such as endogenous proteins or xenobiotics from the external environment. If these toxins enter the brain, they may impair neuronal activity and promote cell death.

The barrier effect of the BBB is mainly formed by ECs. The physical barrier of the BBB, the first barrier effect, refers primarily to the tight junctions between ECs that limit paracellular transport of polar solutes (55). Second, the chemical barrier consists of different transporters for various substances. ECs involved in the BBB contain two types of transporters. One type is nutrient transporters that facilitate the entry of specific nutrients into the CNS and the discharge of specific waste products into the bloodstream (56). The other is efflux transporters, which are generally ATP-binding cassette transporters, such as P-glycoprotein and breast cancer resistance protein (BCRP). Efflux transporters use energy from ATP hydrolysis to pump potentially toxic lipophilic compounds back into the blood and limit the access of many therapeutically used drugs to the brain, providing a mechanism for brain capillary ECs to act as a chemical barrier (57–59). The third barrier effect mediated by ECs is the multienzyme barrier, which may provide the BBB with some degree of substance metabolism capacity (51). Brain capillaries contain a variety of neurotransmitter-metabolizing enzymes, including cholinesterase, GABA transaminase, aminopeptidase, and drug-and toxin-metabolizing enzymes. They protect the brain from circulating neurotransmitters as well as harmful substances with their ability to clear or neutralize (60–62).

## 3 Breakdown of the blood-brain barrier in epilepsy

In earlier studies, it has been proposed that seizures disrupt the normal barrier to transport or diffusion of certain chemicals between the blood and brain tissue. Alternative hypotheses (notably, the BBB hypothesis) in the 19th century have already been used to explain some phenotypes associated with epilepsy (63, 64). Originating from the abnormal physiological state that causes epilepsy, breakage of the BBB loop leads to changes in permeability that can ultimately lead to abnormal entry of substances from small molecules to intact cells (17, 65), which is in turn evidence of microvascular dysfunction in epilepsy (66, 67). Because prolonged opening of the BBB is associated with neuronal network dysfunction and long-term BBB dysfunction itself also indicates cortical damage, it is presumed that BBB damage in the epileptic brain contributes to epilepsy comorbidities. Many animal experiments and instances of clinical evidence also support the hypothesis that vascular lesions, including BBB dysfunction, trigger seizure mechanisms (68). In experimental models of epileptic rats, researchers found that serum IgG leaks into the interstitial space and is taken up by neurons as vascularization progressively increases (69). A process of tight junction protein reduction and increased permeability were also observed in 17-day embryos from the *Vgat^ECKO^* model (70). Notably, vascular remodeling may lead to the formation of leaky vessels, further contributing to epileptogenesis (71). In another rat model of epilepsy, inhibition of angiogenesis by the chemical inhibitor sunitinib was shown to result in seizure freedom (72). Following increased permeability of the BBB, extravasated albumin is taken up into astrocytes (73), resulting in impaired buffering of potassium and glutamate, which may cause neuronal hyperexcitability and spontaneous seizures (74). In addition, activated astrocytes can promote the release of proinflammatory cytokines and chemokines (19). Albumin itself can also be taken up by neurons, leading to hyperexcitability and epileptogenesis (75). Evidence from patients with epilepsy showed that epileptic tissues had significant BBB abnormalities (64) and showed a more significant tendency to hemochemistry than normal tissues (69). Clinically, seizures have been observed in patients with brain lymphoma who underwent a brief BBB opening regimen (76).

### 3.1 Mechanisms of neuronal excitability changes

Under physiological conditions, K^+^ is the major player in neuronal excitation (77). To rapidly repolarize excited neurons, in addition to energy-dependent neuronal mechanisms, a glial buffer is required to achieve stable regulation of K^+^ concentrations under physiological conditions. In particular, astrocyte buffering is responsible for this process (78). It was previously shown that K^+^ is removed from the ECS by glial-specific Na^+^/K^+^ ATPase (79), and it was recently shown that the Na^+^/K^+^/Cl^−^ cotransporter (NKCC1) in astrocytes is associated with BBB disruption (80). Astrocytes can use them to temporarily sequester excess extracellular K^+^ and release it as a buffer when K^+^ levels decline. In addition, astrocytes can also achieve K^+^ homeostasis through spatial buffering. Astrocytes take up K^+^ in areas with high concentrations through gap junctions between adjacent cells and release it in areas with lower concentrations (78, 81). When the K^+^ concentration is high, K^+^ diffuses into the larger extracellular space via net outwards movement guided by the astrocyte network through inwards rectifier potassium channels located in astrocyte foot processes (Kir 4.1) after causing a temporary increase in intracellular concentration (82, 83). The essential role of K^+^ during seizure initiation has been demonstrated. Researchers recorded K^+^ changes in epileptiform activity at different depth positions of the PC and found that surface K^+^ increased during epileptiform activity and spread to the deep layer; they also recorded enhanced spike activity at the same position (84). In animal experiments, extracellular K^+^ concentrations were found to reach the highest levels during irregular firing phases and bursting activity and to eventually recover (85). During seizures, K^+^ transport across the membrane leads to more intense depolarization, action potentials, and increases in potassium flux through potassium channels (86). Drastic membrane depolarization is accompanied by inhibitory collapse of interneurons and allows ictal firing to spread widely in the brain (87). Transient pauses in interneuronal activity may occur under conditions of elevated extracellular K^+^ concentrations and sustain seizure progression by promoting principal cell recruitment (88–90).

Albumin is currently one of the best-known predisposing factors for epilepsy. Albumin itself is not toxic. No neurodegeneration has been observed after intracerebral injection of albumin into mice (91, 92). Following BBB injury, leaked albumin can induce epileptogenesis by lowering the abnormal potential threshold for seizures (93). Albumin has been shown to enhance neural excitability in several studies (18, 73, 74). Albumin uptake by astrocytes is mediated by transforming growth factor, and is followed by downregulation of inwardly rectifying potassium channels, impacting buffering capacity (73). In addition, the absence of aquaporins in the telopodia affects the regulation of water and K^+^, resulting in the disruption of homeostasis of the nervous system. Albumin also attenuates gap junction coupling (94), leading to impaired potassium buffering and increased excitability (95, 96). In addition to changes in neural excitability induced by albumin leakage following BBB disruption, astrocyte uptake of glutamate is also compromised, resulting in loss of excitatory modulation of glutamate buffering assurance (18). BBB damage may also allow zinc in the blood to enter the brain, and its ability to continuously block extrasynaptic GABA-A receptors may lead to neuronal hyperexcitability and seizures (97, 98).

### 3.2 BBB disruption by traumatic brain injury

Traumatic brain injury (TBI) is associated with epilepsy. In Europe and the United States, approximately 2.5 million people develop TBIs each year (99). The risk of seizure development increases dramatically with the severity of the TBI. Seizures develop in 53% of penetrating TBI patients (100). Posttraumatic epilepsy (PTE) is estimated to account for approximately 5% of all cases of epilepsy and 20% of structural epilepsy cases (101). TBI can be categorized according to several dimensions as impingement or nonimpingement; focal or diffuse; and mild, moderate, or severe. Intracranial hemorrhage can be observed in moderate to severe cases. BBB impairment is one of the well-established pathological features of TBI (102–106). Other features include neurovascular structural damage caused by mechanical factors, secondary oedema formation, decreased cerebral perfusion levels, and increased glutamate levels (107–110). BBB damage following most mild TBI in which seizures or epilepsy develop is persistent, and perivascular and distributed IgG staining results have been found in the grey matter of TBI patients who died 1–47 years after initial injury (111, 112). This has also been observed in animal models. Specifically, IgG staining was observed in the corpus callosum 3 months after TBI injury (113). In addition, increased expression of fibronectin and decreased capillary diameter have been observed (114). Cav-1 levels increased in cortical vessels 2 months after injury (115, 116). Finally, TJ proteins such as claudin-5, occludin, and ZO-1 were downregulated during the early post-TBI period; however, levels increased after 1–2 weeks (117, 118). In addition to paracellular alterations, BBB transcellular permeability may also increase after TBI (119). More intuitively interpretable evidence for BBB disruption comes from measurements of vascular endothelial growth factor (VEGF), which is a BBB-related indicator and a protein that regulates angiogenesis and permeability. In animal experiments, increases in VEGF were observed at both 2 h and 1 month following TBI, indicating impaired vascularity in both the short and long term (120, 121). In addition, serum hypoxia-inducible factor 1α (HIF-1α, which regulates VEGF expression) expression was also increased after experimentally induced TBI (122). Neuroimaging further substantiated the disruption of the blood-brain barrier (BBB) in traumatic brain injury (TBI). van Vliet et al. (123) conducted T1-weighted magnetic resonance imaging on an animal model of TBI, revealing diverse levels of contrast agent leakage in the cerebral cortex and thalamus, indicative of notable impairment in BBB function. Notably, even minor trauma can lead to BBB disruption (124). TBI can cause endothelial cell damage by altering tight junction protein expression and the basal lamina, thereby disrupting BBB integrity (125, 126). BBB disruption produced by TBI causes inflammatory responses, lipid peroxidation, and DNA damage, further exacerbating damage to the BBB (126). Oxidative stress has been identified as a major cause of BBB damage in the subacute phase of blast TBI, and subsequent matrix metalloproteinase (MMP) activation can also lead to oxidative stress-mediated loss of BBB integrity (127). During or after TBI, astrocytes and microglia can rapidly respond to injury by affecting BBB function. VEGF, MMP, glutamate, and other substances are specific promoters of BBB integrity loss and are associated with astrocyte activation following TBI (128, 129). Apoptosis and neuroinflammation are also involved in the delayed breakdown of the BBB after TBI (130). Although BBB abnormalities can be observed early after TBI, they usually do not immediately induce seizures, which are observed later in most cases (14, 112).

### 3.3 Neuroinflammation

Inflammation is a physiological process of the body that aims to resist pathogen invasion, is regulated by multiple signals in the immune system and triggers the recruitment of leukocytes. Leukocytes initiate the release of chemotactic cytokines by detecting molecular cues associated with pathogens or injury. Chemotactic cytokines bind to other leukocyte surface receptors and induce local chemotaxis and the accumulation of inflammatory factors. Neuroinflammation is the normal response of the CNS to various insults, such as tissue damage, infection, autoimmune diseases, and epilepsy, and can balance the stronger metabolic needs during increased neuronal activity (131, 132). However, prolonged or excessive neuroinflammatory responses lead to cellular dysfunction in various diseases, including epilepsy. Usually, neuroinflammation is closely associated with dysfunction of the BBB. Various mediators, including inflammatory cytokines and prostaglandins, can affect paracellular TJs and vesicular transport in ECs and are involved in multiple mechanisms (133, 134). Leukocyte transmigration can alter BBB permeability to serum proteins and circulating molecules through adhesion molecule interactions on ECs (63). In the absence of circulating leukocytes, glial-derived interleukin-1 also disrupts the BBB (17). Neuroinflammation and disruption of the BBB are well-established elements of the pathological process of epilepsy following brain injury (135). Gram-negative bacterial lipopolysaccharide (LPS) stimulates macrophages to produce IL-6 and IL-1, two cytokines that increase BBB permeability and are associated with seizure progression (136). LPS also increases BBB permeability and promotes the migration of inflammatory cytokines into the periventricular space (137, 138) as well as an increase in activated microglia (139). Suidan et al. (140) found significant upregulation of VEGF, a classic result of neuroinflammation-induced BBB disruption, in an animal model of neuroinflammation mediated by CD8 T cells. Notably, induction of inflammatory mediators also occurs in BBB ECs, suggesting the progression of inflammation from glia to cerebral microvessels. Inflammatory mediators released by macrophages and granulocytes also enter the brain during epileptogenesis (141). On the one hand, BBB leakage is a consequence of epilepsy. On the other hand, BBB leakage is involved in the promotion of epilepsy progression (15, 142). Epilepsy-related BBB disruption also involves activation of mast cells and mechanisms of action of proinflammatory cytokines (142). The synergistic effect of inflammatory molecules and leukocyte infiltration disrupts the BBB and promotes leakage of serum proteins (68, 143). Increased albumin levels in the brain activate transcriptional signals associated with TGF-β, which can maintain inflammation and induce epileptogenesis (93, 144). Overall, neuroinflammation plays an important role in epileptogenesis, acute symptomatic seizures, and chronic seizures (131, 136). Clinical trials involving positron emission tomography (PET) with translocator protein expressed by activated microglia (TSPO) have demonstrated that seizures, temporal lobe epilepsy, frontal lobe epilepsy, and focal cortical dysplasia are associated with neuroinflammation, and their acute onset leads to chronic neuroinflammation and exacerbates its deterioration (145). Autoimmune diseases increase the risk of epilepsy fourfold in young and middle-aged people (146). Another meta-analysis showed a 2.5-fold increase in the prevalence of epilepsy among patients with systemic autoimmune diseases (SAD) and a 2.5-fold increase in the prevalence of SAD among patients with epilepsy, with a stronger association between SAD and epilepsy in people less than 20 years of age (147). Follow-up studies have found that serum IL-1β and IL-6 levels in the central and peripheral regions of patients with drug-resistant epilepsy decreased at 1 year after surgical treatment compared with those before surgery (148). The remaining studies in patients with epilepsy also demonstrated high serum and CSF levels of multiple proinflammatory cytokines, including IL-6, IL-17, TNF-α, transforming growth factor (TGF)-β, and interferon (IFN)-γ (149–153). Gene expression profiling of surgically resected hippocampi from temporal lobe epilepsy (TLE) patients showed increased levels of chemokines CCL2, CCL3, and CCL4, as well as upregulation of the chemokine receptor CXCR4 (154). Expression levels of the chemokine ligand CX3CL1 were elevated in the hippocampus and adjacent cortex of epileptic rats and in the temporal neocortex of TLE patients (155). Different inflammatory pathways are active in patients with focal epilepsy, including serum nuclear factor kB (NF-kB) (156). In addition, a study by Numis et al. (157) demonstrated that higher levels of proinflammatory cytokines in neonates with encephalopathy were associated with an increased risk of developing epilepsy later in life. Associations between serum levels of inflammatory cytokines and disease course in patients with encephalopathy with status epilepticus during sleep (ESES) have also been demonstrated (153).

Experimental studies further explain the mechanism of the interaction between inflammation and epilepsy. Astrocytes generate many inflammatory molecules, including TGFβ, involved in epilepsy pathogenesis. TGFβ transduction is involved in the degradation of the perineural network around inhibitory neurons and reactive excitatory synapse formation, promoting the recurrence of seizures and pathological neural excitation (158, 159). Correspondingly, as TGFβ signaling blockers, angiotensin receptor 2 antagonists and specific inhibitors prevent functional impairment and reduce seizure frequency in animal models (160, 161). Inflammatory mediators and glutamate produced by astrocytes increase the overexpression of drug transporters in patients with drug-resistant epilepsy (162). In particular, p-glycoprotein reduces the efficacy of various antiepileptic drugs (AEDs) by transporting them from the brain to the blood and restricting their access to the brain (162). Neuroinflammation and oxidative stress were observed to appear before neuronal damage and seizures in mice with progressive myoclonic epilepsy (Unverricht–Lundborg disease) (163). Inflammation exacerbates spiking and wave discharge (SWD) in a rat model of absence epilepsy, a form of epilepsy which can be reduced by anti-inflammatory drugs (162, 164). Endogenous nitric oxide (NO), a proinflammatory mediator, was shown to be a critical factor in seizure initiation and to promote seizures in mice (165). Similarly, HMGB1, another proinflammatory molecule, increased seizure frequency in rats after injection into the hippocampus (166).

Inflammatory processes are also involved in BBB leakage and disruption in status epilepticus. The occurrence of generalized status epilepticus (SE) triggers various inflammatory reactions, such as reactive astrocytosis, microglial activation, and infiltration of mononuclear cells (167). Elevated levels of sphingosine 1-phosphate receptor 1, which contributes to blood-brain barrier permeability in epileptic brains through the promotion of neuroinflammation, were detected alongside reduced levels of tight junction proteins in mice induced with pilocarpine-induced status epilepticus (168). A swift increase in blood-brain barrier permeability within epileptogenic brain regions was also observed during magnetic resonance imaging in this particular animal model (169). This finding serves as crucial evidence linking neuroinflammation to blood-brain barrier dysfunction in status epilepticus. Rojas et al. (170) observed a time-dependent increase in pro-inflammatory mediators and disruption of blood-brain barrier (BBB) integrity in rats with status epilepticus (SE). It is noteworthy that novel pharmacological interventions may be more effective in light of the compromised BBB in SE (171).

### 3.4 The effect of BBB dysfunction on drug therapy

Dysfunction of the BBB mainly impacts drug resistance in epilepsy. Current antiseizure drugs (ASDs) are ineffective in approximately 30% of patients with epilepsy; that is, drug resistance has emerged (172). Drug resistance is associated with increased seizure morbidity and mortality (173). ASDs are often lipophilic and are a potential substrate for BBB efflux carriers, with efflux being an important cause of resistance. P-glycoprotein (Pgp), located on the endothelial luminal membrane of the BBB, is the main efflux carrier of the BBB, and its substrates are estimated to account for 50% of drug candidates (58, 59). Combined ASD is somewhat increased when the BBB is compromised. The drug is transported back into the bloodstream, thereby reducing brain levels of the drug and attenuating its effects (172). For phenytoin in particular, Pgp can reduce brain uptake by up to approximately 70–80% (46). The reversibility of ASD efflux-related epilepsy drug resistance has been experimentally demonstrated. For example, NMDA receptor antagonists or COX-2 inhibitors can inhibit Pgp increase by inhibiting glutamate release during seizures (46, 174). In addition to Pgp, BCRP transports some ASDs, such as lamotrigine. Interestingly, BCRP is expressed at significantly higher levels than Pgp in the human BBB, whereas the opposite is observed in the rodent BBB (175). Increased metabolism of ASDs also leads to increased drug resistance. Cytochrome P450 enzymes, which are responsible for the metabolism of multiple ASDs, have been found to be elevated in the brain ECs of surgically resected tissues from patients with drug-resistant epilepsy (176). In conclusion, BBB leakage in epilepsy does not increase drug levels in the brain parenchyma.

## 4 Molecular biomarkers of epilepsy

### 4.1 Angiogenesis

In neurological diseases, angiogenesis is associated with the permeability of the BBB (177). As a well-known angiogenic factor, VEGF promotes endothelial cell proliferation and migration and increases BBB permeability (178). In medically intractable epilepsy, VEGF regulates aberrant angiogenesis and is upregulated (179, 180). VEGF mediates astrocyte activation and increases seizure-related events (181). Furthermore, VEGF signaling leads to significant epileptiform activity in the hippocampus (179). In animal experiments, sunitinib, a VEGFR-2 inhibitor, completely inhibited pilocarpine-induced angiogenesis and seizures (72). Similarly, rapamycin reduces seizures by inhibiting mTOR, a kinase that mediates positive feedback to the VEGF pathway, which decreases BBB leakage and reduces activation of microglia and macrophages (182). The succinylation of PGK1 disrupts the integrity of the blood-brain barrier by modulating the angiostatin/VEGF pathway, leading to alterations in seizure activity (183). In addition to the members of the VEGF family, multiple factors are also involved in the angiogenic process. EphA4, which can mediate dentate gyrus neurogenesis and angiogenesis, has recently been found to promote angiogenic processes in the CA1 and CA2 regions of the hippocampus (184). The Jagged/Notch1 signaling pathway has been shown to mediate astrocyte regulation of angiogenesis in a mouse model of kainate-induced epilepsy (185).

### 4.2 Inflammation

The upregulation of multiple inflammatory factors upregulated during BBB damage has been demonstrated (186). IL-1R1-TLR4 signaling is involved in initiating neuroinflammatory processes in epilepsy (187). Overactivation of TLR4 and IL-1R1 is associated with autoimmune sepsis, diseases, and neurodegenerative diseases (188–190). In drug-resistant epilepsy, signaling from both receptors stimulates neuronal hyperexcitability (191–194). IL-1R1 and TLR4 are expressed in astrocytes and BBB ECs and have been shown to be upregulated in clinical and experimental studies of epilepsy. IL-1R1 can also be induced in microglia (187). IL-1 receptor-associated kinase 1 (IRAK1) was found to be elevated in temporal lobe epilepsy (195). HMGB1, a ligand of TLR4, can shuttle between the nucleus and cytoplasm. HMGB1, serving as a biomarker for epilepsy, plays a crucial role in initiating neuroinflammation following epileptogenic injury. In TLE and cortical developmental malformations, translocation of HMGB1 impacts upregulation of TLR4 and RAGE (also activated by HMGB1) in neurons and astrocytes (196, 197). In addition, oxidative stress stimulates HMGB1 release, which subsequently promotes seizures by inducing TLR4 and RAGE. This also links neuroinflammation to oxidative stress in epilepsy (198). Recent research has shown that HMGB1 is expressed at markedly elevated levels in the bloodstream of individuals with drug-resistant epilepsy compared to those with well-managed seizures and healthy individuals. This differential expression of HMGB1 suggests its utility as a predictive biomarker for treatment response, holding substantial promise for clinical implementation (199).

Cyclooxygenase 2 (COX2) and arachidonic acid are involved in prostaglandin production. Selective inhibition of COX2 raises the seizure threshold in experimental animals with pentylenetetrazole-induced acute transient epilepsy. However, this effect was not observed in rodents with status epilepticus (200). In experiments in rats with pilocarpine-induced status epilepticus, a reduction in the number and severity of chronic seizures was observed following treatment with celecoxib or parecoxib, COX2 inhibiting drugs (201, 202). Aspirin, as a nonselective COX1 and COX2 inhibitor, has also been investigated in epilepsy-related trials. In animal experiments, brief aspirin treatment reduced the frequency and duration of seizures and was associated with neuroprotective effects (203).

### 4.3 Oxidative stress

Oxidative stress can act as an important link between angiogenesis and inflammation and has been demonstrated to be associated with multiple neurological diseases, such as stroke, traumatic brain injury, Alzheimer’s disease, Parkinson’s disease, Huntington’s disease, and multiple sclerosis. Oxidative stress exacerbates BBB disruption through activation of the inducible enzymes NADPH oxidase 4 (NOX4) and NOX5. It also increases levels of inflammatory cell infiltration in the nervous system (204, 205). Nuclear factor erythroid 2-related factor (Nrf2) is also a regulator of oxidative stress, involved through the transcription of antioxidant detoxification genes (206). Nrf2 has been demonstrated to affect BBB integrity by regulating the expression of BBB endothelial TJ proteins such as ZO-1 and OCLN (207). Pathological oxidative stress can cause the destruction of reactive free radicals and the BBB, which inevitably affects the progression of epilepsy. The inhibition of reactive oxygen species (ROS) and reactive nitrogen species (RNS), as representative intracellular signaling molecules during oxidative stress, have been shown to ameliorate cognitive deficits caused by epilepsy and improve associated survival (208–210). In a rat model of epilepsy, seizure progression was suppressed after the application of antioxidants, and seizure frequency was drastically reduced after discontinuation of drug administration by blocking ROS production (196). In another similar animal trial, the antioxidant treatment promoted the translocation of the transcription factor NRF2 to the nucleus and gene transcription of antioxidant enzymes, ultimately blocking epilepsy progression and producing neuroprotective effects (211). Notably, NRF2 is a key molecule involved in the treatment of epilepsy with antioxidants. Induced expression of NRF2 in the mouse hippocampus reduced seizure frequency and duration. In addition, NRF2 was shown to directly regulate 3.5% of differentially expressed genes between the hippocampus of epileptic patients and controls (212).

## 5 Other BBB-related neurological disorders

The BBB is associated not only with epilepsy but also with many other neurological diseases. The mechanisms underlying BBB damage in these diseases are similar, which not only demonstrates the importance of the BBB but may also be an important clue for the development of treatments for such diseases in the future.

### 5.1 Alzheimer’s disease

Alzheimer’s disease (AD), discovered 100 years ago by German physician Dr. Alois Alzheimer (213), is a disease characterized by impaired cognition and memory due to neuronal vulnerability (214). AD is characterized pathologically by the accumulation of hyperphosphorylated tau-forming neurofibrillary tangles (NFTs) in the brain and accumulation of amyloid-β (Aβ) into plaques (215). The progression of AD is associated with BBB dysfunction, even at an early stage (216, 217). In addition to a significantly higher rate of tracer leakage (218), tau oligomers and Aβ were found to accumulate in the cerebral vessels of AD model mice (219), and eventually the mice developed cognitive decline. Neuroimaging revealed that BBB permeability was significantly increased in the gray matter of patients with early-stage AD compared with controls (220). Moreover, promotion of brain tissue infiltration by peripheral macrophages by disruption of the BBB has also been found to be exacerbated in AD patients upon autopsy (221, 222).

There are many similarities in the mechanisms underlying BBB disruption between epilepsy and AD. For example, as in epilepsy, neuroinflammation is an important mechanism of BBB disruption in AD patients. Proinflammatory cytokines produced by microglia and astrocytes upon activation cause upregulation of MMP-9 expression in endothelial cells, which results in the degradation of tight junction proteins and causes the breakdown of the BBB (223). In turn, increased permeability of the BBB promotes immune cells to enter brain tissue, which is normally prevented by the BBB, and increases neuroinflammation (224). Infiltration of immune cells into the central nervous system can affect neurological function. T cells in the aging brain inhibit the proliferation of neural stem cells (225), and cytokines that mediate the infiltration process are also thought to have an important relationship with cognitive impairment (226).

Aβ and tau are classical markers of AD, and disruption of BBB also worsens AD by causing their deposition. In addition to transporting antiepileptic drug as described above, Pgp can transport Aβ out of the brain (227). Glucose transporter 1 (GLUT1) also promotes Aβ clearance from the brain via the LRP1-dependent pathway (228). Studies have confirmed that GLUT1 and Pgp are expressed at reduced levels in endothelial cells of cerebral vessels in AD patients, resulting in the accumulation of Aβ in the brain by disrupting its clearance (229, 230). ABCC1 demonstrates a comparable and notably influential role in the pathogenesis of AD. In an animal model of AD expressing ABCC1, the activation of this protein substantially decreased the accumulation of Aβ (231). Furthermore, the overexpression of ABCC1 resulted in a reduction in Aβ production and an increase in the cleavage ratio of amyloid precursor protein (232).

Tau forms NFTs, which are a hallmark of AD. Some studies have revealed that tau can cause leakage of the BBB even in the absence of Aβ (233). In aged AD model mice, tau is overexpressed, the cerebral microvessels are disrupted, and abnormal malformed vascular masses form, and cortical atrophy occurs (234). Tau expression is also negatively correlated with cerebral blood flow, a decrease in which is often accompanied by cognitive deterioration (235).

BBB disruption is a common pathological hallmark of AD and epilepsy (236). Indeed, epilepsy is frequently a comorbidity of AD. Emerging evidence suggests that early-stage AD patients are also at risk for epilepsy, and this proportion is not low (237, 238). Compared with patients with AD alone, AD patients with epilepsy not only have an earlier onset of cognitive decline, but also have a shorter life expectancy (239–241). This demonstrates that epilepsy can accelerate the deterioration of AD patients’ condition. The BBB deserves further investigation as a point of connection between the two neurological diseases.

### 5.2 Multiple sclerosis

Multiple sclerosis is a chronic inflammatory demyelinating disease of the CNS (242). There are approximately 2.3 million people with MS worldwide (243). MS is an autoimmune disease in which reactive T cells interact with antigens presented by macrophages or microglia under pathological conditions, resulting in damage to myelin sheaths and axons and ultimately neuronal loss (244–246). Cytokines secreted by activated macrophages can also interfere with myelination and the expression of related genes (247), preventing myelin sheath repair and causing disability (248). Similar to epilepsy pathogenesis, disruption and leakage of the BBB are important contributors to MS pathogenesis. Fibrinogen deposition was observed following T-cell infiltration in demyelinated lesions, indicating increased endothelial permeability (249). TJ abnormalities, including claudin-3 loss, are also found in progressive MS, suggesting pathological opening of paracellular channels (250, 251). In fact, BBB changes often appear in the early stages of MS, before the formation of active lesions, which are a significant feature of early-stage MS (252, 253). Recent findings have even shown that functional abnormalities of the BBB represent additional pathological changes associated with MS (254). Because of the association of MS with the immune system, impaired neuroimmunity is often involved in MS-related BBB damage (255).

Endothelial cells under pathological conditions up-regulate membrane receptor expression and promote the release of pro-inflammatory factors from glial cells, increasing the recruitment of leukocytes to the CNS (256, 257). Reactive astrocytes can also separate the ends from capillary endothelial cells (258). These neuroimmune responses disrupt the TJ and endothelial cell membranes, allowing the BBB to continuously break down and increase its leakage.

### 5.3 Amyotrophic lateral sclerosis

Amyotrophic lateral sclerosis (ALS) is one of the most common motor neuron diseases. This progressive neurodegenerative disease leads to eventual death due to paralysis and respiratory failure. ALS usually occurs in the middle-aged and elderly population, with a prevalence of 4.1–8.4 per 100,000 people, and is characterized by progressive loss of upper and lower motor neurons at the spinal cord or medullary level (259, 260). Disruption of the BBB or blood-spinal cord barrier (BSCB) is one of the features of ALS. Leakage of Evans blue in spinal capillaries was observed in an earlier mouse model of ALS (261). Detected downregulation of CD146 and laminin in mice revealed disruption of the endothelium and basement membrane (262). Erythrocytes were even observed in the spinal cord (263). In addition, previous studies have found BBB disruption due to MMP-9 activation in patients and animal models (264), and the decrease levels of tight junction protein and adhesions protein in spinal cord (265). Autopsy results also confirmed dramatic reduction in pericyte numbers (266).

Interestingly, similar to epilepsy, both neuroinflammation and oxidative stress are associated with progressive impairment of the BBB/BSCS in ALS. Activation of microglia, resulting in the release of proinflammatory cytokines and the recruitment of astrocytes, has been observed in animal models of ALS (267). Proinflammatory factors can impair tight junction protein expression. Inhibition of IL and TNF signaling in mice has been found to prolong their lifespan (268). Astrocytes exacerbate neurodegeneration after they are activated and impair vascular repair and vasculature formation. In addition, researchers have found that mutations in the angiopoietin gene are associated with neuroinflammation and disrupted BBB integrity (269). In addition to marked neuroinflammation, increased levels of ROS and decreased levels of antioxidants such as reduced glutathione have been detected in the cerebrospinal fluid and blood of ALS patients (270), implying the important role of oxidative stress in ALS development. ROS are involved in glutamate transport disorders in astrocytes, increasing neuronal excitotoxicity and promoting ALS development (271).

## 6 Conclusion

The occurrence of seizures and the advancement of epilepsy are intricately linked to the functionality of the blood-brain barrier. Various factors contribute to or worsen seizures by compromising the integrity of the blood-brain barrier. Additionally, seizures in cases of severe epilepsy can further enhance the permeability of the blood-brain barrier. The neural activity within the brain is impacted by a multitude of factors that are transported through the bloodstream. The BBB, acting as a protective barrier between the blood and the nervous system, holds significant potential in the regulation of epilepsy. In our study, we summarized relevant biomarkers that could potentially be utilized as targets for future research in understanding the mechanisms of epilepsy and developing more effective antiepileptic medications.

## Author contributions

JH: Visualization, Writing – original draft. YW: Conceptualization, Methodology, Supervision, Writing – review & editing. PW: Project administration, Supervision, Writing – review & editing. DL: Methodology, Project administration, Visualization, Writing – original draft. YS: Funding acquisition, Resources, Supervision, Writing – review & editing.
